# P-1345. Risk Factors for Ceftriaxone Resistant *E. coli* or *K. pneumoniae* Infections in Floor Patients

**DOI:** 10.1093/ofid/ofae631.1522

**Published:** 2025-01-29

**Authors:** Holly Westerkamp, Olivia Knack, Jenna Adams, Fritzie S Albarillo

**Affiliations:** Duke University Hospital, Palatine, Illinois; Loyola University Medical Center, Maywood, Illinois; University of Illinois Chicago, Chicago, Illinois; Loyola University Medical Center, Maywood, Illinois

## Abstract

**Background:**

In 2019, 1.3 million deaths worldwide were linked to antimicrobial resistant organisms. Resistant organisms are associated with poor outcomes including increased mortality and length of stay (LOS). Identification of local risk factors predisposing for resistant organisms can lead to rapid antimicrobial optimization.

Patient Population

**Figure:**
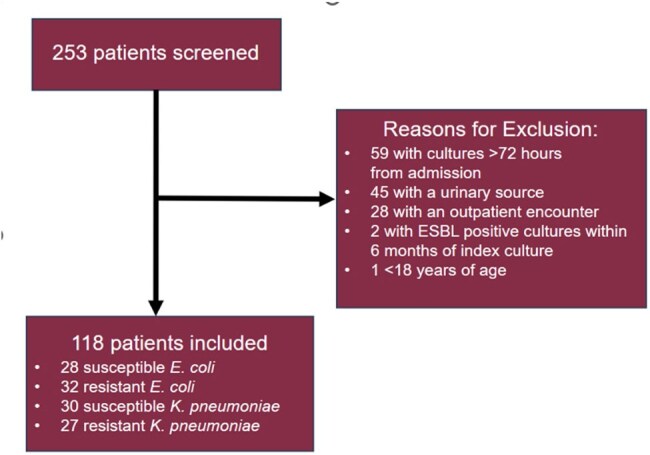

**Methods:**

In this retrospective, single center, observational cohort study, floor patients with ceftriaxone (CRO) resistant *E. coli* or *K. pneumoniae* via broth microdilution were screened from January 2022 to October 2023. Exclusion criteria included urine isolates, extended spectrum beta-lactamase (ESBL) or CRO resistant *E. coli* or *K. pneumoniae* isolate within 6 months of index culture, and intensive care unit (ICU) admission at time of index culture. The primary outcome was to identify risk factors for CRO resistant *E. coli* and *K. pneumoniae* in floor patients. Secondary outcomes included 14-day all-cause mortality, ICU admission rate, hospital LOS, and 30-day readmission rate. A list of patients was generated via MedMined™. REDCap™ was used for data collection. All statistical analyses were performed using IBM SPSS™. Significance was defined at *p<* 0.05.

Multivariate Logistical Regression

**Figure:**
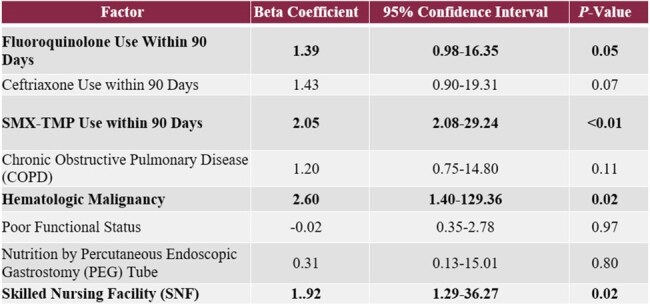

**Results:**

118 patients were included in the statistical analyses. The following risk factors found to be statistically significant were: fluoroquinolone (FQ) (*p*< 0.01), ceftriaxone (CRO) (*p<* 0.01), or sulfamethoxazole-trimethoprim (SMX-TMP) (*p*< 0.01) use within 90-days of index culture; chronic obstructive pulmonary disease (*p*=0.01); ventilator or tracheostomy use at admission (*p*=0.03); active hematologic malignancy (*p*=0.01); poor functional status at admission (*p*=0.02); percutaneous endoscopic gastrostomy tube at admission (*p*=0.01); admission from a skilled nursing facility (SNF) (*p*=0.01). The significant risk factors were included in the multivariate logistic regression. FQ use (95% CI 0.98-16.35, *p*=0.05), SMX-TMP use (95% CI 2.08-29.24, *p*< 0.01), hematologic malignancy (95% CI 1.40-129.36, *p*=0.02), and admission from a SNF (95% CI 1.29-36.27, *p*=0.02) were found to meet statistical significance.

**Conclusion:**

FQ use and SMX-TMP use within 90-days of index culture, hematologic malignancy, and admission from a SNF were found to be independently associated with ceftriaxone resistance.

**Disclosures:**

**All Authors**: No reported disclosures

